# Aflatoxin Exposure May Contribute to Chronic Hepatomegaly in Kenyan School Children

**DOI:** 10.1289/ehp.1104357

**Published:** 2012-02-27

**Authors:** Yun Yun Gong, Shona Wilson, Joseph K. Mwatha, Michael N. Routledge, Jovita M. Castelino, Bin Zhao, Gachuhi Kimani, H. Curtis Kariuki, Birgitte J. Vennervald, David W. Dunne, Christopher P. Wild

**Affiliations:** 1 Division of Epidemiology, Leeds Institute for Genetics, Health and Therapeutics, University of Leeds, Leeds, United Kingdom; 2 Department of Pathology, University of Cambridge, Cambridge, United Kingdom; 3 Kenya Medical Research Institute, Nairobi, Kenya; 4 Division of Vector Borne Diseases, Ministry of Health, Nairobi, Kenya; 5 DBL-Centre for Health Research and Development, Faculty of Life Science, University of Copenhagen, Copenhagen, Denmark; 6 International Agency for Research on Cancer, Lyon, France

**Keywords:** aflatoxicosis outbreak, aflatoxin, aflatoxin albumin adducts, biomarker, child health, hepatomegaly, hepatosplenomegaly, malaria, schistosomiasis

## Abstract

Background: Presentation with a firm type of chronic hepatomegaly of multifactorial etiology is common among school-age children in sub-Saharan Africa.

Objective: Aflatoxin is a liver toxin and carcinogen contaminating staple maize food. In this study we examined its role in chronic hepatomegaly.

Methods: Plasma samples collected in 2002 and again in 2004 from 218 children attending two schools in neighboring villages were assayed for aflatoxin exposure using the aflatoxin-albumin adduct (AF-alb) biomarker. Data were previously examined for associations among hepatomegaly, malaria, and schistosomiasis.

Results: AF-alb levels were high in children from both schools, but the geometric mean (95% confidence interval) in year 2002 was significantly higher in Matangini [206.5 (175.5, 243.0) pg/mg albumin] than in Yumbuni [73.2 (61.6, 87.0) pg/mg; *p* < 0.001]. AF-alb levels also were higher in children with firm hepatomegaly [176.6 (129.6, 240.7) pg/mg] than in normal children [79.9 (49.6, 128.7) pg/mg; *p* = 0.029]. After adjusting for *Schistosoma mansoni* and *Plasmodium* infection, we estimated a significant 43% increase in the prevalence of hepatomegaly/hepatosplenomegaly for every natural-log-unit increase in AF-alb. In 2004, AF-alb levels were markedly higher than in 2002 [539.7 (463.3, 628.7) vs. 114.5 (99.7, 131.4) pg/mg; *p* < 0.001] but with no significant difference between the villages or between hepatomegaly and normal groups [539.7 (436.7, 666.9) vs. 512.6 (297.3, 883.8) pg/mg], possibly because acute exposures during an aflatoxicosis outbreak in 2004 may have masked any potential underlying relationship.

Conclusions: Exposure to aflatoxin was associated with childhood chronic hepatomegaly in 2002. These preliminary data suggest an additional health risk that may be related to aflatoxin exposure in children, a hypothesis that merits further testing.

In sub-Saharan Africa, presentation with chronic hepatomegaly (HM) that usually has a firm to hard consistency upon palpation is common among school-age children (Gryseels 1988). Enlargement of the liver can be extensive and associated with accompanying enlargement of the spleen [splenomegaly (SM)], which is also of a firm to hard consistency upon palpation (Fulford et al. 1991). Long-term health outcomes of this condition are yet to be defined, but it is likely to have an adverse impact on health. It is associated with increases in portal pressure (Vennervald et al. 2004), and recent analysis suggested an association with slowed child growth (Wilson et al. 2010). Chronic hepatosplenomegaly (HSM) has a complex etiology of a multifactorial nature. Among school-age children in Makueni District, Kenya, where chronic HSM is highly prevalent, chronic exposure to malaria infection has been shown to be associated with HSM, with a particularly strong association with enlargement of the spleen being observed (Mwatha et al. 2003; Wilson et al. 2007b). It has also been shown that concurrent *Schistosoma mansoni* infection exacerbates this underlying HSM (Booth et al. 2004b; Wilson et al. 2007b). Although these two infections appear to contribute to HSM, less than satisfactory resolution after treatment of the infections suggests that other etiological factors may contribute to the HM observed in these children.

Aflatoxin, a secondary metabolite produced by *Aspergillus* species, is a liver toxicant and a known human liver carcinogen (International Agency for Research on Cancer 2002). The hot and humid weather in sub-Saharan Africa is favorable to fungal growth and aflatoxin production. Aflatoxin exposure has been consistently found at high levels in sub-Saharan African populations because of consumption of highly contaminated food such as maize and groundnuts (Wild and Gong 2010). Many children are chronically exposed to high levels of aflatoxin through diet, and the exposure is associated with child growth impairment in West Africa (Gong et al. 2002, 2003, 2004). The consequences of such early-life exposure merits more investigation to assess the full extent of the impact on child health.

High levels of contamination and exposure to aflatoxin have been reported in Kenya, including in the Makueni District (Lewis et al. 2005; Mwihia et al. 2008; Ngindu et al. 1982). One outbreak occurred in 1981 (Ngindu et al. 1982), during which 20 patients with jaundice were hospitalized and 12 died of hepatic failure because of consumption of maize heavily contaminated with aflatoxin. In 2004, 317 cases of acute hepatic failure due to ingestion of highly aflatoxin-contaminated locally grown maize, including 125 deaths, were reported across several districts, including Makueni (Azziz-Baumgartner et al. 2005; Strosnider et al. 2006). The aflatoxin level in the market maize samples was up to 46,400 ppb, more than a thousand-fold higher than the regulated 20-ppb maximum limit in food; 65% of the maize samples from Makueni were above the 20-ppb limit (Lewis et al. 2005). At the same time, levels of aflatoxin–albumin adducts (AF-alb), an aflatoxin exposure biomarker, were the highest ever reported (McCoy et al. 2008; Wild and Gong 2010), ranging up to 67,000 pg/mg albumin.

Here we provide a preliminary analysis of the association between HM and aflatoxin exposure during 2002 and 2004 in a cohort of school-age children living in Makueni District.

## Methods

*Study area and population.* Makueni District is characterized by seasonal rains typically falling in November–December and April–May. The study population was based in two neighboring village schools: Matangini Primary in Lower Mangelete in the east, and Yumbuni Primary in the west. Lower Mangelete has a series of permanent streams and irrigational canals, whereas surface water in Yumbuni is mainly seasonal. The main dietary staples in the population are the maize-based dishes githeri and ugali. A detailed description of the study area is given elsewhere (Wilson et al. 2007a).

We conducted a cross-sectional school-based study in May–June 2002, including all children 6–17 years of age who were attending the Matangini (*n* = 228) and Yumbuni (*n* = 221) primary schools at the time of the study. Most of the children attended their local village school, so we use the name of the school attended (Matangini and Yumbuni) to represent the village in which the child lived. A few children attending the schools who were > 17 years old were excluded from the study.

In all children, malaria infection was determined by parasitological techniques, including measurement of parasitemia and *Plasmodium falciparum* schizont antigen IgG3 (Pfs-IgG3) levels, a marker of chronic exposure to *Plasmodium* infections (Wilson et al. 2007a). Schistosomiasis was diagnosed based on the *S. mansoni* egg count from three separate stool samples, using the Kato Katz technique; two slides were prepared from each sample, as described elsewhere (Wilson et al. 2007b).

A subset of 249 children was randomly selected for ultrasound examination and blood sampling in 2002 and with a repeat examination in 2004. AF-alb levels were measured for all children in this subset except a small number of individuals (*n* = 31) whose blood samples were exhausted in previous immunological assays that had been applied to all children in the subset. Age, sex, and health were not significantly different between the wider cohort and the subset of children (data not shown).

Informed consent was obtained from the parents or guardians. Ethics approval was obtained from the Kenya Medical Research Institute Ethical Review Committee.

*Clinical examination.* All children were examined for enlarged livers and spleens by palpation by three clinicians in 2002 and 2004, and a consensus among the clinicians had to be achieved before assigning the child to an organomegaly group, as previously described (Wilson et al. 2007b). The liver and/or spleen was considered enlarged if it was palpable > 2 cm below the costal line. The children were initially classified into five categories: no organomegaly, soft liver or spleen enlargement, firm to hard spleen enlargement (SM), firm to hard liver enlargement (HM), and both firm to hard liver and spleen enlargement (HSM).

*Blood AF-alb measurements.* Plasma samples collected from the 218 children examined in both 2002 and 2004 underwent AF-alb analysis in 2009. The blood AF-alb levels were determined using an enzyme-linked immunosorbent assay (ELISA) after albumin extraction from 250 μL plasma, digestion, and purification as previously described (Chapot and Wild 1991). One negative and three positive controls were analyzed alongside each batch of samples. Samples were measured using ELISA in quadruplicate on at least two occasions on separate days. The detection limit was 3 pg AF-alb per 1 mg albumin.

*Statistical analyses.* AF-alb data is not normally distributed and was therefore natural-log-transformed before data analysis. Geometric mean AF-alb levels and 95% confidence intervals (CIs) are presented unless otherwise stated. Student’s *t*-test and analysis of variance (ANOVA) were used for comparing levels between groups. Chi-square test was used for categorical variables distribution test. Pearson’s correlation coefficients were calculated for the association between continuous variables. Logistic regression models were constructed to estimate odds ratios for groupings of HM in association with aflatoxin exposure adjusted for schistosomiasis and malaria, which are known confounders or modifiers. Statistical analyses were carried out using STATA (version 9; StataCorp, College Station, TX, USA). A *p*-value < 0.05 was used to define statistical significance.

## Results

*Description of the study cohort.* Table 1 summarizes the key variables of the 124 and 94 children 6–17 years of age from Yumbuni and Matangini. There were no significant differences in mean age or sex ratio between the two schools. Malaria and schistosomiasis were prevalent in this region. In 2002, the prevalence of schistosomiasis in these children was significantly higher in Matangini than in Yumbuni (70% vs. 8%, *p* < 0.001). There was no significant difference in the prevalence of malaria based on the presence of parasitemia (27% vs. 18%, *p* = 0.159) or the Pfs-IgG3 marker of exposure [optical density (OD) value, 0.47 vs. 0.44; *p* = 0.106] between children attending the two schools.

**Table 1:** Description of the cohort in 2002 (except as specified).

School		
Variable	Yumbuni	Matangini	Total
n		124		94		218
Age [years (mean ± SD)]		11.6 ± 3.0		12.5 ± 2.8		12.0 ± 2.9
Male:female ratio		1:1		1:1		1:1
Malaria prevalence		20/109 (18%)		24/90 (27%)		44/199 (22%)
S. mansoni prevalence		10/124 (8%)		66/94 (70%)*		76/218 (35%)
Pfs-IgG3 [OD (mean ± SD)]		0.44 ± 0.15		0.47 ± 0.13		0.45 ± 0.10
AF-alb [geometric mean (95% CI)]						
2002		73.2 (61.6, 87.0)		206.5 (175.5, 243.0)*		114.5 (99.7, 131.4)
2004		578.5 (466.4, 717.6)		492.0 (397.3, 609.2)		539.7 (463.3, 628.7)
*p ≤ 0.001 compared with Yumbuni.						

*Aflatoxin exposure.* Plasma AF-alb levels were not correlated with the children’s age or sex. The geometric mean AF-alb level for children in Matangini was significantly higher than that of children in Yumbuni [206.5 pg/mg (175.7, 243.0) vs. 73.2 pg/mg (61.6, 87.0) pg/mg; *p* < 0.001] in 2002 (Table 1). During 2004, AF-alb mean levels were significantly higher than in 2002 in both villages [combined means of 539.7 pg/mg (463.3, 628.7) in 2004 vs. 114.5 pg/mg (99.7, 131.4) in 2002; *p* < 0.001], but there was no longer a significant difference in the means between the two villages.

AF-alb levels were not associated with malaria parasitemia or correlated with Pfs-IgG3 levels. Children with *S. mansoni* infection had more than double the AF-alb level as those without in 2002. However, the difference became nonsignificant after data were adjusted for village. There was no significant association between *S. mansoni* infection and AF-alb in 2004 (data not shown).

*Prevalence of HM.* The prevalence of HM/HSM was not significantly different between Yumbuni and Matangini in 2002 (73% vs. 76%; *p* = 0.686) or 2004 (74% vs. 83%; *p* = 0.087). There was no significant difference in HM prevalence between the subset and the wider group of the cohort (data not shown).

The number of children with HM only was 48 in year 2002, but this increased to 104 in 2004 (Table 2). Most children (88%) who had HM/HSM in 2002 still had HM/HSM in 2004. Most new HM cases in 2004 had been classified as HSM cases in 2002 but had resolution of their SM. The total number of children with HM/HSM did not vary significantly between 2002 and 2004 (160 vs. 167).

**Table 2:** AF-alb (pg/mg) in different organomegaly groups in 2002 and 2004 [geometric mean (95% CI)].

2002			2004		
Organomegaly group	na	AF-alb	na	AF-alb
Normal		21		79.9 (49.6, 128.7)*		35		512.6 (297.3, 883.8)
Soft organomegaly		13		57.5 (27.7, 119.2)*		2		452.9b
SM		21		102.6 (65.4, 160.8)		12		604.3 (332.9, 1096.7)
HM		48		176.6 (129.6, 240.7)		104		539.7 (436.7, 666.9)
HSM		112		108.0 (90.5, 129.0)		63		533.7 (416.4, 684.0)
aThree children in 2002 and one child in 2004 were not classifiable into one of the clinical groups, and one child in 2004 had missing AF-alb data; they were all excluded. bThere is no CI because only two children were in this group. *p < 0.05 compared with the HM group by ANOVA.								

There was no significant relationship between the presentation of organomegaly and the age or the sex of the children in 2002 (data not shown). However, in 2004 children with HM/HSM (mean age, 11.5 years) were younger than normal children and children with SM (mean age, 13.5 years), whereas the two children with soft organomegaly were the youngest in the group (mean age, 6.5 years). More girls were in the normal group than boys in 2004 (22% vs. 12%; *p* = 0.041).

*Aflatoxin exposure and organomegaly.* In 2002, the geometric mean AF-alb level was significantly higher in children with HM than in children without firm organomegaly (Table 2)—that is, children classified as normal or with soft organomegaly (*p* = 0.029 and 0.004, respectively). No significant difference in geometric mean AF-alb levels was found among HM, SM, and HSM groups [176.6 pg/mg (129.6, 240.7), 102.6 pg/mg (65.4, 160.8), and 108.0 pg/mg (90.5, 129.0), respectively]. For samples collected in 2004, AF-alb levels were markedly high in all groups, and no significant difference in AF-alb levels was observed between children with HM and any other groups.

After adjusting for *S. mansoni* infection and Pfs-IgG3 quartiles, which had previously been shown to be associated with HSM in the wider cohort, there was a 43% increase in the prevalence of HM/HSM versus non-HM for each natural log unit increase in AF-alb (odds ratio = 1.43; 95% CI: 1.04, 1.97) (Table 3).

**Table 3:** Logistic regression model for HM/HSM in 2002.

Variable	Odds ratio (95% CI)	p-Value
ln(AF-alb)		1.43 (1.04, 1.97)		0.030
S. mansoni infectiona		1.58 (0.73, 3.40)		0.241
Pfs-IgG3		1.20 (0.90, 1.60)		0.215
aAs determined by the presence of detectable eggs.				

## Discussion

Childhood HSM in sub-Saharan Africa is likely to be of a complex multifactorial etiology. In the present study, samples collected during a previous study among school-age children in 2002 and at follow-up in 2004 were used to examine associations between HSM and aflatoxin exposure using the AF-alb biomarker.

The AF-alb biomarker has a relatively long half-life (1–2 months) compared with other aflatoxin biomarkers. It is well accepted to be a reliable marker in aflatoxin exposure assessment and has been applied to studies of chronic diseases (Wild and Gong 2010), including carcinogenesis, and growth retardation and immune-suppression effects in young children (Gong et al. 2002, 2004; Wild and Gong 2010). The biomarker has been repeatedly applied in retrospective type studies (Wild, and Gong 2010) and there is no evidence that this biomarker is liable to degradation in stored plasma samples.

Compared with exposure in other populations in sub-Saharan Africa (Wild and Gong 2010), aflatoxin exposure was already high in 2002, especially in Matangini, with the exposure level nearly triple that of children from Yumbuni. Initial analysis in 2002 indicated that there was a significant association between increased exposure to aflatoxin and *S. mansoni* infection, with *S. mansoni* infection being more prevalent and AF-alb levels being elevated in Matangini compared with Yumbuni. The presence of permanent surface water makes Matangini more humid than Yumbuni and hence more favorable to fungal growth and aflatoxin production compared with Yumbuni. The permanent streams and irrigation canals in Matangini also provide a habitat for *Biomphalaria* species, the aquatic snail that is the intermediate host of *S. mansoni*, whereas the lack of permanent surface water abrogates transmission in Yumbuni. The association between *S. mansoni* infection and AF-alb levels is therefore likely attributable to environmental conditions in Matangini being more favorable than in Yumbuni for both *S. mansoni* transmission and growth of *Aspergillus* species. This is substantiated by the reduced magnitude of association when comparing AF-alb levels between children with or without *S. mansoni* infection when adjusted for location. However, the biologically plausible hypothesis that aflatoxin exposure may induce chronic liver damage, which increases the susceptibility to schistosomiasis-associated morbidity—or vice versa, that the liver damage due to schistosomiasis infection may alter the metabolism of aflatoxin and also the synthesis of albumin, which could affect the AF-alb level—merits further analysis in studies designed to address this question.

In contrast to a previous report (Allen et al. 1992), the AF-alb level did not differ significantly between children with or without malaria parasitemia, nor was it associated with the chronic malaria marker Pfs-IgG3 in the present study. Future study on the direct relationship between aflatoxin exposure and malaria is required.

The AF-alb levels were exceptionally high in 2004—in fact the highest ever reported—and the difference between villages was not observed. This exceptional level is consistent with the widely reported aflatoxicosis outbreak in 2004 in Kenya when AF-alb levels were the highest ever published (McCoy et al. 2008). The reason for the outbreak was consumption of home-grown maize that was highly contaminated with aflatoxin because of both the drought stress and being stored at warmer indoor conditions after the poor harvest, to prevent theft (Azziz-Baumgartner et al. 2005). It was also reported that the contaminated home-grown maize was sold in the local market and was bought by farmers after their home-grown supplies were exhausted, resulting in widespread distribution of the contaminated maize (Lewis et al. 2005). Village location played a less significant role in the AF-alb levels during the outbreak.

Aflatoxin is a liver toxin in several animal species (International Agency for Research on Cancer 2002). It causes liver enlargement in broiler chickens (Kumar and Balachandran 2009; Mani et al. 2000) and in animals suffering aflatoxicosis (Gonzalez Pereyra et al. 2008; Osman et al. 2004). To our knowledge there are no previous reports of chronic HM occurrence in chronically exposed humans. In 2002, the AF-alb levels were significantly higher in children with HM than in those without firm organomegaly, indicating that aflatoxin exposure may play an important role in HM occurrence. The nonsignificant difference in the AF-alb level between HSM and other groups suggests that the etiology of HSM may be even more complex than HM, so contribution by aflatoxin exposure could be diluted. For example, the effect of malaria exposure is greater on the spleen than on the liver, and vice versa for schistosomiasis, and there is evidence that both infections contribute to enlargement of both organs (Wilson et al. 2007b). After controlling for *S. mansoni* and *Plasmodium* infection, there was an association between aflatoxin and HM, with the prevalence of having HM (including HSM) being increased by 43% in association with a natural-log-unit increase in AF-alb. The mechanism needs further elucidation, although compromised liver function and the subsequent changes in liver metabolism due to chronic exposure to aflatoxin may contribute to HM development.

*S. mansoni* infection status and Pfs-IgG3 were not significantly associated with HM/HSM in the logistic model that also included AF-alb. In the wider cohort, *S. mansoni* was associated with an increase in the size of the liver, suggesting this infection could exacerbate the condition, but there was no statistically significant association with prevalence of HM (Wilson et al. 2007b). Also in the wider cohort, increased exposure to *Plasmodium* infection did show a weak association with HM only, but the levels of the Pfs-IgG3 were not elevated in HM as much as in HSM (Wilson et al. 2007b). In a previous study in Makueni District, annual treatment of *S. mansoni* infections and regular mollusciding of the River Kambu (the habitat for the intermediate host) over a 3-year period did not achieve complete resolution of childhood HM (Vennervald et al. 2005). In that study the children who lived close to the river and had the highest levels of Pfs-IgG3 had the poorest resolution of their HM (Booth et al. 2004a). All this evidence suggests that the HM/HSM observed in these children is of a chronic complex nature and may also be reversible in a slow process.

During the interim period between the 2002 and 2004 examinations, there was a substantial increase in HM cases, largely due to a shift from HSM to HM—in other words, a reduction in SM cases. These represent individual children whose condition changed from HSM to HM during the 2-year period; that is, the HM status persisted, showing that this was a chronic condition in these children, whereas SM was reversed. The reduction in SM cases may possibly be explained by reduced malaria transmission due to the drought that was associated with the acute aflatoxin outbreak in 2004, which would also have had a significant impact on the seasonal transmission of malaria, as evidenced by a drop in Pfs-IgG3 levels (Wilson S, unpublished observations).

The aflatoxicosis outbreak in 2004 resulted in extremely high levels of AF-alb in most of the children, and there was no significant association between AF-alb and HM/HSM in 2004. It is possible that this lack of association in 2004 between HM and AF-alb may result from the high acute exposure during the aflatoxicosis outbreak masking any putative underlying relationship between aflatoxin exposure and chronic HM. It does not in itself negate the potential association between the two, but it is recognized that the association observed in 2002 may be a false positive and therefore requires investigation in other studies.

A large-scale association study is now desirable, specifically designed to address the role of aflatoxin in HM and taking account of the role of infections. Alternatively, an intervention study, if feasible, would provide the best approach to draw a conclusion about the etiological association between HM and aflatoxin exposure. Nevertheless, the present study highlights a potential new health effect of aflatoxin exposure. This further emphasizes the need for education to improve public awareness of aflatoxin-associated health risks and for the development of effective aflatoxin prevention strategies. It is worth noting that the need for intervention has already been identified in this region of Kenya and is being put into practice, which may have contributed to the reduced severity of the 2010 aflatoxicosis outbreak.

## Conclusion

This study raises the possibility of an association between aflatoxin exposure and childhood HM, at least at exposure levels that do not induce acute aflatoxicosis. This finding provides the first evidence pointing to this health effect of these liver toxicants. This observation merits further study in other areas of high aflatoxin exposure, in studies designed to directly test this hypothesis.

## References

[r1] Allen SJ, Wild CP, Wheeler JG, Riley EM, Montesano R, Bennett S (1992). Aflatoxin exposure, malaria and hepatitis-B infection in rural Gambian children.. Trans R Soc Trop Med Hyg.

[r2] Azziz-Baumgartner E, Lindblade K, Gieseker K, Rogers HS, Kieszak S, Njapau H (2005). Case-control study of an acute aflatoxicosis outbreak, Kenya, 2004.. Environ Health Perspect.

[r3] Booth M Vennervald BJ Butterworth AE Kariuki HC Amaganga C Kimani G 2004a Exposure to malaria affects the regression of hepatosplenomegaly after treatment for *Schistosoma mansoni* infection in Kenyan children. BMC Med 2 36; doi:10.1186/1741-7015-2-36 [Online 27 September 2004] 15450118 PMC522803

[r4] Booth M Vennervald BJ Kenty LC Butterworth AE Kariuki HC Kadzo H 2004b Micro-geographical variation in exposure to *Schistosoma mansoni* and malaria, and exacerbation of splenomegaly in Kenyan school-aged children. BMC Infect Dis 4 13; doi:10.1186/1471-2334-4-13 [Online 17 May 2004] 15147584 PMC421731

[r5] Chapot B, Wild CP (1991). ELISA for quantification of aflatoxin-albumin adducts and their application to human exposure assessment.

[r6] Fulford AJ, Mbugua GG, Ouma JH, Kariuki HC, Sturrock RF, Butterworth AE (1991). Differences in the rate of hepatosplenomegaly due to Schistosoma mansoni infection between two areas in Machakos District, Kenya.. Trans R Soc Trop Med Hyg.

[r7] Gong YY, Cardwell K, Hounsa A, Egal S, Turner PC, Hall AJ (2002). Dietary aflatoxin exposure and impaired growth in young children from Benin and Togo: cross sectional study.. BMJ.

[r8] Gong YY, Egal S, Hounsa A, Turner PC, Hall AJ, Cardwell KF (2003). Determinants of aflatoxin exposure in young children from Benin and Togo, West Africa: the critical role of weaning.. Int J Epidemiol.

[r9] Gong YY, Hounsa A, Egal S, Turner PC, Sutcliffe AE, Cardwell K (2004). Post-weaning exposure to aflatoxin results in impaired child growth: a longitudinal study in Benin, West Africa.. Environ Health Perspect.

[r10] Gonzalez Pereyra ML, Carvalho ECQ, Tissera JL, Keller KM, Magnoli CE, Rosa CAR (2008). An outbreak of acute aflatoxicosis on a chinchilla (*Chinchilla lanigera*) farm in Argentina.. J Vet Diagn Invest.

[r11] Gryseels B. (1988). The morbidity of schistosomiasis mansoni in the Rusizi Plain (Burundi).. Trans R Soc Trop Med Hyg.

[r12] International Agency for Research on Cancer (2002). Some traditional herbal medicines, some mycotoxins, naphthalene and styrene.. IARC Monogr Eval Carcinog Risks Hum.

[r13] Kumar R, Balachandran C. (2009). Histopathological changes in broiler chickens fed aflatoxin and cyclopiazonic acid.. Vet Arhiv.

[r14] Lewis L, Onsongo M, Njapau H, Schurz-Rogers H, Luber G, Kieszak S (2005). Aflatoxin contamination of commercial maize products during an outbreak of acute aflatoxicosis in eastern and central Kenya.. Environ Health Perspect.

[r15] Mani K, Sundaresan K, Viswanathan K. (2000). Performance of commercial broiler strains under experimental aflatoxicosis.. Indian J Poultry Sci.

[r16] McCoy LF, Scholl PF, Sutcliffe AE, Kieszak SM, Powers CD, Rogers HS (2008). Human aflatoxin albumin adducts quantitatively compared by ELISA, HPLC with fluorescence detection, and HPLC with isotope dilution mass spectrometry.. Cancer Epidemiol Biomarkers Prev.

[r17] Mwatha JK, Jones FM, Mohamed G, Naus CW, Riley EM (2003). Associations between anti-*Schistosoma mansoni* and anti-*Plasmodium falciparum* antibody responses and hepatosplenomegaly in Kenya schoolchildren.. J Infect Dis.

[r18] Mwihia JT, Straetmans M, Ibrahim A, Njau J, Muhenje O, Guracha A (2008). Aflatoxin levels in locally grown maize from Makueni District, Kenya.. East Afr Med J.

[r19] Ngindu A, Kenya PR, Ocheng DM, Omondi TN, Ngare W, Gatei D (1982). Outbreak of acute hepatitis caused by aflatoxin poisoning in Kenya.. Lancet.

[r20] Osman N, El-Sabban FF, Al Khawli A, Mensah-Brown EPK (2004). Effect of foodstuff contamination by aflatoxin on the one-humped camel (*Camelus dromedarius*) in Al Ain, United Arab Emirates.. Aust Vet J.

[r21] Strosnider H, Azziz-Baumgartner E, Banziger M, Bhat RV, Breiman R, Brune MN (2006). Workgroup report: public health strategies for reducing aflatoxin exposure in developing countries.. Environ Health Perspect.

[r22] Vennervald BJ, Booth M, Butterworth AE, Kariuki HC, Kadzo H, Ireri E (2005). Regression of hepatosplenomegaly in Kenyan school-age children after praziquantel treatment and three years of greatly reduced exposure to *Schistosoma mansoni*.. Trans R Soc Trop Med Hyg.

[r23] Vennervald BJ, Kenty LC, Butterworth AE, Kariuki CH, Kadzo H, Amaganga C (2004). Detailed clinical and ultrasound examination of children and adolescents in a *Schistosoma mansoni* endemic area in Kenya: hepatosplenic disease in the absence of portal fibrosis.. Trop Med Int Health.

[r24] Wild CP, Gong YY (2010). Mycotoxins and human disease: a largely ignored global health issue.. Carcinogenesis.

[r25] Wilson S Booth M Jones FM Mwatha JK Kimani G Kariuki HC 2007a Age-adjusted *Plasmodium falciparum* antibody levels in school-age children are a stable marker of microgeographical variation in exposure in exposure to Plasmodium infection. BMC Infect Dis 7 67; doi:10.1186/1471-2334-7-67 [Online 29 June 2007] 17603885 PMC1947991

[r26] Wilson S, Vennervald BJ, Kadzo H, Ireri E, Amaganga C, Booth M (2007b). hepatosplenomegaly in Kenyan schoolchildren: exacerbation by concurrent chronic exposure to malaria and *Schistosoma mansoni* infection.. Trop Med Int Health.

[r27] Wilson S, Vennervald BJ, Kadzo H, Ireri E, Amaganga C, Booth M (2010). Health implications of chronic hepatosplenomegaly in Kenyan school-aged children chronically exposed to malarial infections and *Schistosoma mansoni*.. Trans R Soc Trop Med Hyg.

